# Characterisation of a Desmosterol Reductase Involved in Phytosterol Dealkylation in the Silkworm, *Bombyx mori*

**DOI:** 10.1371/journal.pone.0021316

**Published:** 2011-06-27

**Authors:** Leonora F. Ciufo, Patricia A. Murray, Anu Thompson, Daniel J. Rigden, Huw H. Rees

**Affiliations:** 1 Institute of Integrative Biology, University of Liverpool, Liverpool, United Kingdom; 2 School of Environmental Sciences, University of Liverpool, Liverpool, United Kingdom; New Mexico State University, United States of America

## Abstract

Most species of invertebrate animals cannot synthesise sterols *de novo* and many that feed on plants dealkylate phytosterols (mostly C_29_ and C_28_) yielding cholesterol (C_27_). The final step of this dealkylation pathway involves desmosterol reductase (DHCR24)-catalysed reduction of desmosterol to cholesterol. We now report the molecular characterisation in the silkworm, *Bombyx mori*, of such a desmosterol reductase involved in production of cholesterol from phytosterol, rather than in *de novo* synthesis of cholesterol. Phylogenomic analysis of putative desmosterol reductases revealed the occurrence of various clades that allowed for the identification of a strong reductase candidate gene in *Bombyx mori* (BGIBMGA 005735). Following PCR-based cloning of the cDNA (1.6 kb) and its heterologous expression in *Saccharomyces cerevisae*, the recombinant protein catalysed reduction of desmosterol to cholesterol in an NADH- and FAD- dependent reaction.

Conceptual translation of the cDNA, that encodes a 58.9 kDa protein, and database searching, revealed that the enzyme belongs to an FAD-dependent oxidoreductase family. Western blotting revealed reductase protein expression exclusively in the microsomal subcellular fraction and primarily in the gut. The protein is peripherally associated with microsomal membranes. 2D-native gel and PAGE analysis revealed that the reductase is part of a large complex with molecular weight approximately 250kDa. The protein occurs in midgut microsomes at a fairly constant level throughout development in the last two instars, but is drastically reduced during the wandering stage in preparation for metamorphosis. Putative Broad Complex transcription factor-binding sites detectable upstream of the DHCR24 gene may play a role in this down-regulation.

## Introduction

Most species of invertebrates are incapable of synthesis of sterols *de novo* and rely on a dietary source of these compounds [1]. Many of these species, such as plant pest insects and plant-parasitic nematodes, obtain primarily C_29_ and C_28_ sterols (e.g. sitosterol and campesterol) from plants. However, such sterols, unprocessed, cannot satisfy the sterol requirement for normal growth and development of many of these invertebrates, which have a specific need for a C_27_ sterol, such as cholesterol [2], [3]. Thus, many invertebrate species dealkylate phytosterols obtained from the plant, yielding cholesterol or a closely related ring-modified C_27_ sterol [3]. The invertebrate phyla/species which have been demonstrated to be capable of phytosterol dealkylation, include phytophagous insect species [2], [3], yellow fever mosquito, *Aedes aegypti* [4], an insect-parasitic [5] and certain free-living nematodes, such as *Caenorhabditis elegans* [6]–[8] and *Turbatrix aceti* [9], some Crustacea, Coelenterates, Molluscs [10]–[12], and a protozoan [13]. In the case of plant-parasitic nematodes, owing to the difficulty of culturing them outside their host plant, it has not been possible to directly investigate phytosterol dealkylation by radiolabelling experiments. However, by comparison of the sterol composition of the nematodes with the host plants, good evidence exists for the occurrence of dealkylation in *Ditylenchus dipsaci* [14] and *Rotylenchulus reniformis* [15]. In contrast, whereas the results of such an approach for *Heterodera zeae* [16], *Meloidogyne incognita* and *M. arenaria* [17] are suggestive of the existence of C-24 dealkylation in these species, they are inconclusive owing to the possibility of selective uptake of cholesterol from the diet [18], [19].

**Figure 1:**
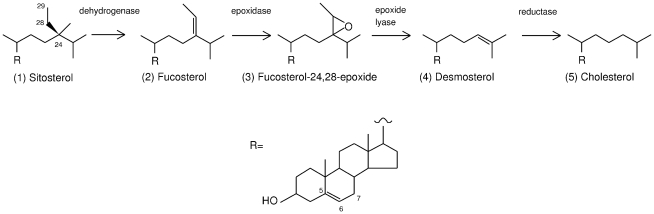
Pathway of transformation of sitosterol into cholesterol.

The majority of work on characterization of the phytosterol dealkylation pathways has been undertaken in phytophagous insects, owing to their greater size [2], [3], [20]. However, the transformations occurring have also been delineated in detail in the model nematode, *C. elegans* [6]–[8]. In plants, the most commonly occurring major sterol is probably sitosterol (C_29_), frequently accompanied by lesser amounts of campesterol (C_28_) and the 22-unsaturated stigmasterol (C_29_). It has been established that the steps in dealkylation of each of these sterols are essentially analogous [2], [3], [20], although whether a single enzyme catalyzes the equivalent reactions in each case or separate enzymes exist for the reactions of different sterols is uncertain. The pathway from sitosterol (1) to cholesterol (5) is shown in Fig. 1 and involves dehydrogenation of sitosterol to yield fucosterol (2), which undergoes epoxidation to fucosterol-24(28)-epoxide (3). Epoxide lyase-catalysed cleavage (dealkylation) of this product gives desmosterol (4), which undergoes desmosterol reductase-catalysed reduction to cholesterol (5). The side chain dealkylation steps in *C. elegans* are also the same as in insects [8]. All the foregoing steps of dealkylation have been demonstrated to occur in insect midgut and, with the exception of the first step, have also been achieved *in vitro* using microsomal (endoplasmic reticulum) preparations [2]. In both insects and *C. elegans*, the sterol nucleus may also undergo various transformations. The phytosterol dealkylation pathway has clear potential for development of novel pest insect and nematode control strategies.

The desmosterol reductase in vertebrate species catalyzes the reduction of desmosterol to cholesterol, as one of the two potential final steps in the *de novo* cholesterol biosynthetic pathways [21]. We now report the cloning, recombinant expression and characterization of the cDNA encoding desmosterol reductase from the silkworm, *Bombyx mori*, together with characterization of the corresponding protein. This is the first characterised desmosterol reductase enzyme not involved in *de novo* sterol biosynthesis, as in vertebrates, but instead in the transformation (dealkylation) of C_29_ and C_28_ plant sterols into C_27_ sterols.

## Materials and Methods

All chemicals were obtained from Sigma.

### Bioinformatic analysis

A putative desmosterol reductase gene sequence for *Bombyx mori* was obtained by the following bioinformatic procedure. The human 3β-hydroxysterol Δ^24^ reductase (DHCR24) sequence [22] was used to search *B. mori* protein sequences at Silkdb (http://silkworm.genomics.org.cn/) with BLAST [23]. This identified two homologues with identifiers BGIBMGA005735 and BGIBMGA012624. An alignment of the human and the two potential *B. mori* sequences was produced using Clustal X. Each homologue was used to further query the nr database [24] and sequences of close relatives retrieved. These were supplemented with sequences obtained at genome resource webpages for *Meloidogyne incognita* (http://www.inra.fr/meloidogyne_incognita/) [25] and *M. hapla* (http://www.pngg.org/cbnp/) [26]. Since relatives of the Arabidopsis DIMINUTO gene product were not the prime focus of this work only a selection of these was included. These, along with a more distantly related plant cytokinin dehydrogenase to serve as an outgroup in phylogenetic analysis, were aligned with MUSCLE [27]. Phylogenetic analysis was done with MEGA 4 [28] as follows. The evolutionary history was inferred using the Minimum Evolution method [29] employing 500 replicates generated by bootstrapping [30]. The evolutionary distances were computed using the p-distance method [31] and are in the units of the number of base differences per site. The ME tree was searched using the Close-Neighbor-Interchange (CNI) algorithm [31] at a search level of 1 and the Neighbor-joining algorithm [32] was used to generate the initial tree. All positions containing gaps and missing data were eliminated. Subcellular localization was predicted using TargetP [33]. The 5′ upstream regions of candidate *B. mori* genes were searched against the TRANSFAC database [34] at http://www.biobase.de.

### Cloning of *B. mori* cDNA

Total RNA was extracted from the midgut of day 5, 5th instar *B. mori* larvae. First strand DNA was synthesised using a Bioline cDNA kit. Double-stranded DNA was generated using Pfu polymerase (Stratagene) from the first strand DNA in a PCR reaction using the primers CCCCAAGCTTATGGCCATAGAAACAGAAACGTTTTTGG and CCCCGGATCCTTATTTCCTAACATTTCTGTTGACTTTATCATAGATCTCGG. The PCR product was ligated to a galactose-inducible yeast expression vector pYES2 (Invitrogen) using the BamHI and HindIII sites included in the primers. The plasmid was verified by sequencing the coding sequence and the junctions with the plasmid.

### Expression in yeast

Yeast cells were propagated using YEPD medium [1% (w/v) yeast extract, 1% (w/v) peptone, 2% (w/v) glucose] and yeast transformants were grown in minimal medium [0.67% (w/v) yeast nitrogen base (without amino acids), 2% (w/v) glucose, galactose or raffinose as alternative carbon sources and 1.92 g/l yeast synthetic drop-out medium without uracil]. All media were obtained from Sigma. The yeast strain BY4741 [Mata his3Δ1 leu2Δ0 met15Δ0 ura3Δ0] was transformed with the plasmid construct [35]. After purification of colonies on minimal glucose medium, cultures were grown on minimal raffinose medium overnight to remove residual glucose which would interfere with galactose induction. Minimal galactose medium was inoculated with the overnight cultures at a low OD_600_ of approximately 0.05 and grown shaking at 30°C to an OD_600_ of 1. Cultures were harvested, the cell pellet washed and resuspended in a minimal amount of 20 mM Tris.HCl pH 7.5, 50 mM NaCl and lysed by vortexing with glass beads for 4×1 min with 2 min cooling intervals on ice. The total yeast homogenate was removed from the beads and stored in single use aliquots at −80°C.

### Measurement of DHCR24 activity in yeast cell extracts

The yeast homogenates were assayed for protein concentration and phosphoglucose isomerase activity as in Waterham *et al.* [22] to allow for normalisation between the various extracts used. Reactions were analysed in triplicate from yeast extracts made from experimental transformants and also from yeast transformed with the pYES2 vector alone as a negative control. The assay mixture contained 100 mM Tris.HCl pH 7.23, 0.1 mM dithiothreitol, 30 mM nicotinamide, 3.5 mM nicotinamide adenine dinucleotide phosphate, 30 mM glucose-6-phosphate, 2 U/ml glucose-6-phosphate dehydrogenase [for generation of reduced nicotinamide adenine dinucleotide phosphate (NADPH)], 0.5 mg/ml bovine serum albumin and 168 µM desmosterol (prepared as 420 µM stock in 1.25% methyl-β-cyclodextrin, Tris.HCl pH 7.23). Requirement for FAD and NADPH was assessed by leaving these components out of the assay mixture. In the case of NADPH, the regenerating system was also left out of the assay mixture. The assays were incubated for 4 h at 37°C and then terminated by adding 2 ml 50% methanol/2 M KOH, mixed well and left to saponify the lipids and sterol esters overnight at room temperature. Sterols were extracted with 3×2 ml hexane washes which were pooled and then washed with 10 ml H_2_O to reduce the salt concentration and pH. The hexane wash was then dried completely under nitrogen, derivatised with N,O-bis(trimethylsilyl)trifluoroacetamide, dried again before finally dissolving in 100 µl dichloromethane and analysed using a ThermoQuest CE gas chromatograph (Trace 2000 series) coupled with a Thermo-Finnigan TSQ-7000 mass spectrometer. The GC was fitted with an on-column injector and a capillary column (DB5-MS; 60 m×0.25 mm i.d., 0.10 µm film thickness, which was fitted with a 1 m retention gap, J & W Scientific, CA, USA). The oven was initially held at 60°C for 1 min, then heated from 60 to180°C at 12°C min^−1^ and from 180 to 315°C at 2.5°C min^−1^, and held for 10 min at 315°C. Helium was used as carrier gas at a constant flow (1.6 ml min^−1^, with vacuum compensation). A stream of air was used to cool the injector prior to, and for 1 min after each injection.

Typical operating conditions for mass spectrometry were: electron energy at 70eV, scanning from 50 to 600 Thomsons, scan time of 1 s, ion source temperature at 230°C , interface temperature at 320°C. The emission current was set to 300 µA and the multiplier at 1200 V. Xcalibur Software (Version 1.0) was used to acquire and process the data. Sterol peaks were quantified by comparison of their peak areas with those of 5α-cholestane included (15 µg) as an internal standard in the triplicate reactions. Standard mixtures containing this quantity of 5α-cholestane and 0.5–15 µg desmosterol after derivatisation were also dissolved in 200 µl dichloromethane and analysed by GC/MS to produce a calibration curve.

### Growth and dissection of insects

*B. mori* eggs [four-way polyhybrid strain (126×57)×(70×79)] and artificial diet were obtained from Professor Silvia Cappellozza, CRA- Unità di Ricerca di Api-Bachcioltura, sede di Padova, 35143 Padova, Italy. Eggs were incubated at 27°C for approximately 5 days prior to hatching and then kept at 27°C and fed on artificial diet [36].The larvae progress through all developmental instars in 30 days under these conditions. Insects were taken from various stages of growth and immobilized by chilling on ice before dissecting midgut and various other tissues. The tissues were kept on ice in buffer I (37 mM HEPES/NaOH, pH 7, 300 mM sucrose) during the dissection procedure. The midguts were sliced longitudinally and rinsed repeatedly in buffer I to remove gut contents.

### Preparation of microsomes

Larval tissues were resuspended in a minimal volume of buffer I and homogenised in a Potter- Elvehjem mechanical homogeniser with 10 strokes. The homogenate was then centrifuged at 1000 g and the supernatant collected. The pellet (mainly cell debris) was resuspended in buffer I, re-homogenised, centrifuged as before and the supernatant pooled with the previous one to increase yield of protein. The supernatant was then centrifuged at 12000 g for 20 min and the supernatant collected and subjected to a final centrifugation at 150000 g for 90 min to yield the crude microsomal pellet and the soluble supernatant.

### Antibody production

Antibodies were raised by Genscript Corporation using the peptide sequence, IFNNPGQLKIKPGE, chosen using their software (residues 405–418 of the predicted sequence of the *Bombyx* DHCR24 protein, located towards the C- terminal end).

### Western blot analysis

The subcellular fractions of larval tissues were normalised for protein content and resuspended in SDS PAGE sample buffer and heated to denature proteins before separating on 10% SDS PAGE gels and transferring to nitrocellulose. The nitrocellulose was incubated in 5% milk powder dissolved in TBS (20 mM Tris.HCl pH 7.4, 150 mM NaCl) for 1 h before incubating with antibodies at a dilution of 1∶1000 in the same milk solution overnight at 4°C. The nitrocellulose membrane was then washed thoroughly with TBS, incubated with anti-rabbit horseradish peroxidase antibodies and the signal developed with a chemiluminescence solution (100 mM Tris.HCl pH 8.5, 1.25 mM luminol, 0.2 mM p-coumaric acid, 0.01% H_2_O_2_) and exposed to light-sensitive film.

### Alkaline sodium carbonate treatment

Analysis of location of *B. mori* DHCR24 on microsomes (endoplasmic reticulum) was carried out essentially as described in Fujiki *et al.* [37]. Briefly, microsomes were resuspended in ten volumes of alkaline solution (0.1 M Na_2_CO_3_ pH 11.5) and incubated for 30 min on ice before re-isolating microsomes by centrifugation and analysing their DHCR24 content by immunoblotting.

### Blue native gels

Native blue gel analysis [38] was carried out using the Invitrogen blue gel solutions and Novex 4–16% gradient gels. Samples were prepared according to the manufacturer's instructions using 12 µg of crude microsome protein per lane on the gel. Briefly, microsomes were dissolved in 10% dodecylmaltoside on ice for 30 min. Sample buffer and G250 additive were added and samples run on 3–12% Novex Bis-Tris gels. At the end of the first dimension, lanes from the Bis-Tris gel were soaked in 10 mM ß-mercaptoethanol and 1% SDS for 45 minutes before assembling the strip at the top of a 12% standard SDS-PAGE gel and separating in the second dimension. The second dimension gel was then used for western blot analysis.

## Results

### Phylogenetic analysis

**Figure 2:**
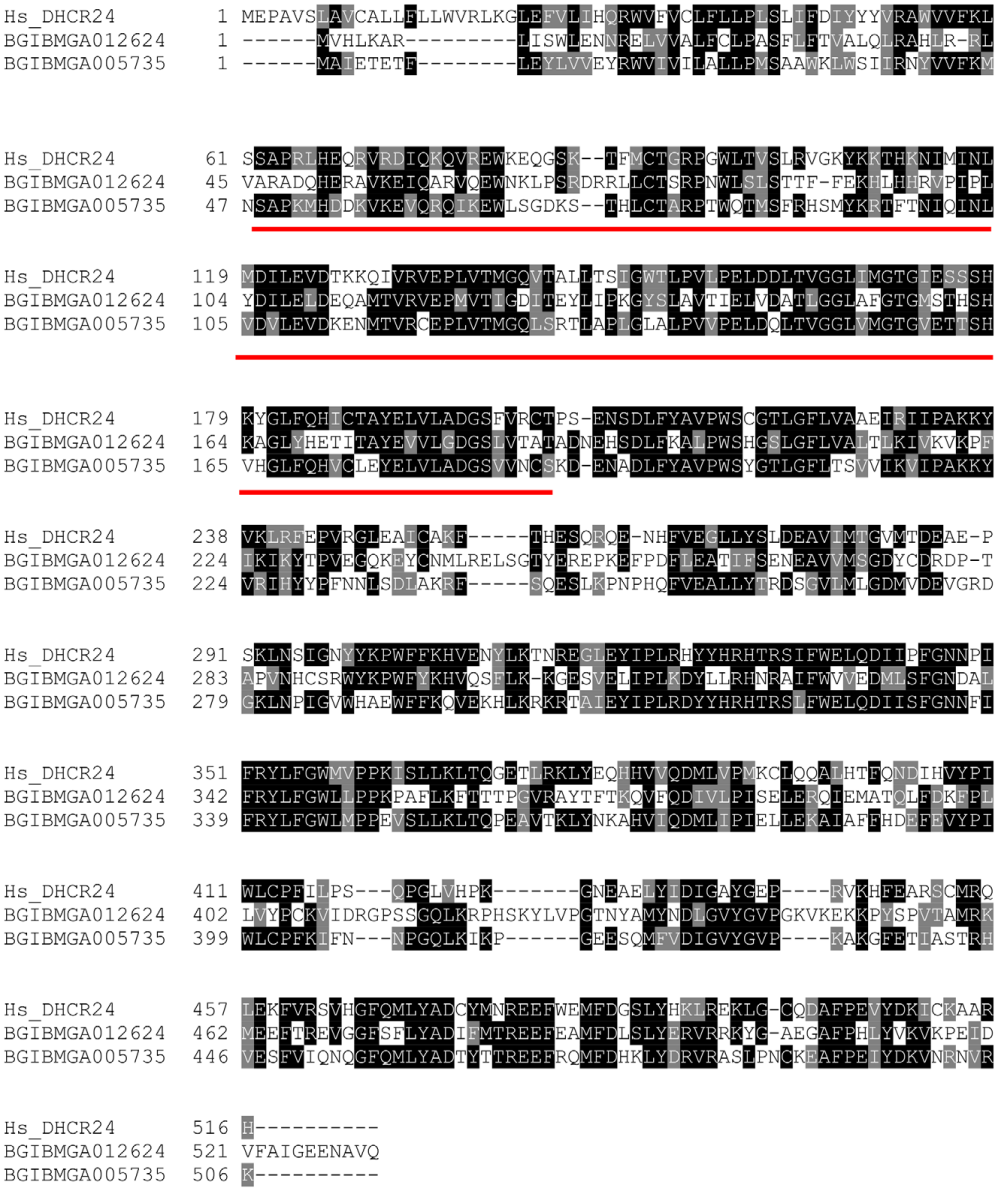
Alignment of human DHCR24 with *B. mori* proteins studied here. Similarities are shaded in pale grey, identities are shaded in dark grey. The region matching the Pfam [39] entry for a FAD-binding domain is underlined.

**Figure 3:**
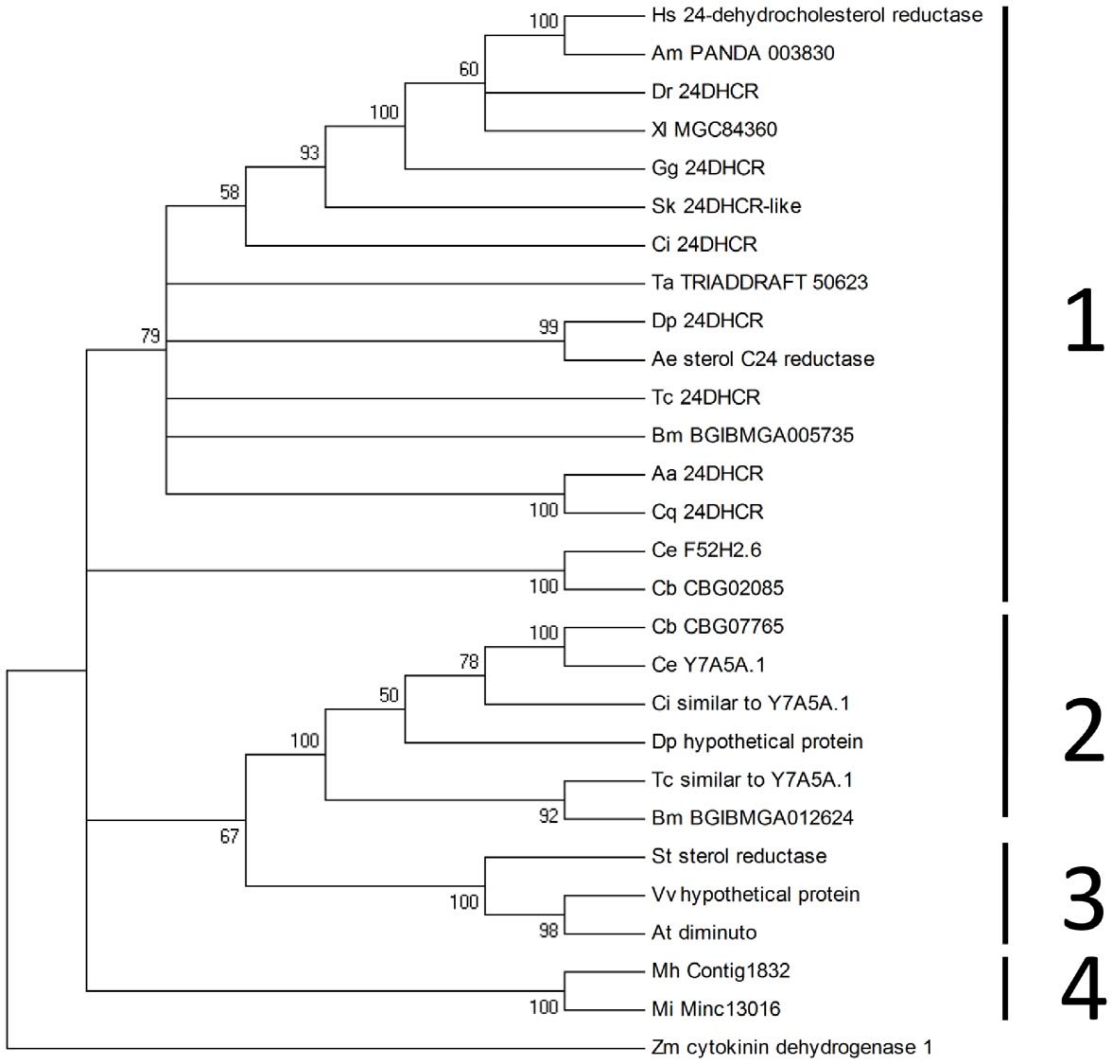
Phylogenetic analysis. Sequences of DHCR24s and related proteins were subjected to phylogenetic analysis by the Minimum Evolution method with MEGA4 [28] as detailed in Materials and Methods. For display, branches corresponding to partitions reproduced in less than 50% bootstrap replicates are collapsed: for the remaining branches, percentage bootstrap values are shown next to the branches [30]. Well-supported clades discussed in the text are labelled 1 to 4. The other sequences are Aa_24DHCR (gi:157131548, *Aedes aegypti*); Aa_”24DHCR” (gi:157131550, *A. aegypti*); Tc_24DHCR (gi:189233852, *Tribolium castaneum*); Cq_24DHCR (gi:170028705, *Culex quinquefasciatus*); Dp_24DHCR (gi:251825191, *Daphnia pulex*); Gg_24DHCR (gi:71896815, *Gallus gallus*); Ci_24DHCR (gi:198436362, *Ciona intestinalis*); Sk_24DHCR-like (gi:291242187, *Saccoglossus kowalevskii*); Dr_24DHCR (gi:56693363, *Danio rerio*), Am_PANDA_003830 (gi:281353588, *Ailuropoda melanoleuca*); Tc_similar_to_Y7A5A.1 (gi:91094133, *Tribolium castaneum*); Dp_hypothetical_protein (gi:251825189, *Daphnia pulex*); Ci_similar_to_Y7A5A.1 (gi:198414872, *Ciona intestinalis*); Ce_Y7A5A.1 (gi:17570305, *Caenorhabditis elegans*); Ce_F52H2.6 (gi:17568041, *C. elegans*) Cb_CBG07765 (gi:268581995, *Caenorhabditis briggsae*); Cb_CBG02085 (gi:268577743, *C. briggsae*); Xl_MGC84360 (gi:148231947, *Xenopus laevis*); Hs_24DHCR (gi:13375618, *Homo sapiens*); Ta_TRIADDRAFT_50623 (gi:196010403, *Trichoplax adhaerens*); Ae_sterol_C24_reductase (gi:189026985 completed – see text; *Aphanomyces euteiches*); At_diminuto (gi:602302, *Arabidopsis thaliana*); St_sterol_reductase (gi:302127800, *Solanum tuberosum*); Vv_hypothetical_protein (gi:147861641, *Vitis vinifera*); Mh_Contig_1832 (*Meloidogyne hapla*); Mi_Minc13016 (*Meloidogyne incognita*); Zm_cytokinin_dehydrogenase_1 (gi:162462431, *Zea mays*). The sequence of maize cytokinin dehydrogenase was used as an outgroup.

An alignment of the human DHCR24 protein and the two *B. mori* sequences is shown in Fig. 2. The human sequence showed 52% and 38% sequence identities to BGIBMGA005735 and BGIBMGA012624, respectively. Both sequences match Pfam [39] domain FAD_binding_4 (PF01565) indicating their membership of a family of FAD-dependent oxidoreductases. In order to assess the relationship of the two *B. mori* sequences with others of known or unknown function in the databases, phylogenetic analysis was done with MEGA 4 [28]. Fig. 3 shows a bootstrapped Minimum Evolution tree where nodes supported in less than half of trees have been collapsed. Major features of the tree are also found in Neighbour Joining and Maximum Parsimony trees (data not shown). Using the more distantly related cytokinin dehydrogenase as an outgroup, phylogenetic analysis divides the sequences shown into four distinct groups. BGIBMGA005735 and BGIBMGA012624 cluster with group 1 and group 2 sequences, respectively.

### Cloning and recombinant expression of a putative *B. mori* DHCR24 cDNA

**Figure 4:**
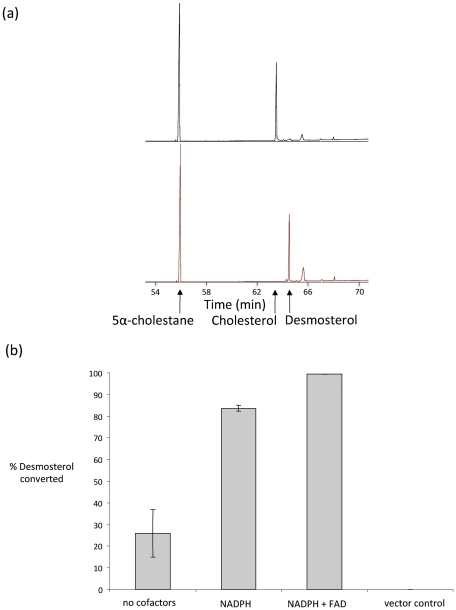
DHCR24 activity of *B. mori* BGIBMGA005735 expressed in *S. cerevisiae*. Yeast homogenates containing expressed *B. mori* BGIBMGA005735 were added to assay mixtures containing desmosterol and various combinations of the cofactors NADPH and FAD. A yeast homogenate containing expressed pYES2 (vector control) was incubated with both cofactors as a negative control. Assays were incubated for 4 h at 37°C and products analysed by GC/MS. MS trace (total ion current) of positive reaction containing cholesterol and negative reaction containing only desmosterol are shown in Fig. 4a. Sterol peaks were calibrated using triplicate reactions containing 5α-cholestane as an internal standard and expressed as a percentage of desmosterol converted into cholesterol compared to the empty vector reaction (Fig 4b). The positions of elution of authentic cholesterol and desmosterol are shown by arrows (Fig. 4a).

Since BGIBMGA005735 bore a closer phylogenetic relationship (group 1) to other desmosterol reductases than BGIBMGA012624 (group 2), and also had appreciably higher sequence identity to human DHC24 than BGIBMGA012624, attention was focused on the former. Specific primers to the sequence BGIBMGA005735 were used to amplify the DNA from *B. mori* midgut cDNA and the DNA was cloned into pYES2, a galactose-inducible yeast expression vector. Cells expressing the construct were processed to produce a yeast cell extract and used in an assay to determine the presence of activity for conversion of desmosterol into cholesterol. *S. cerevisiae* does not have enzymes capable of carrying out this conversion so background levels in the empty vector control were zero (Fig. 4b). In contrast, the cell extract containing the expressed construct converts all of the desmosterol added into cholesterol in the time frame of the assay in the presence of NADPH and FAD (4 h; Fig. 4b). The conversion to cholesterol was in fact found to be complete in less than 1 hour in these assay conditions (data not shown). The identity of the desmosterol and cholesterol peaks was verified by GC-MS of the trimethylsilyl derivatives (desmosterol, m/z 456[M]^+^, 441, 366, 343, 327, 253, 129); cholesterol, m/z 458[M]^+^, 368, 353, 329, 129; both spectra were in agreement with those of authentic samples [40]. The enzyme will, therefore, be referred to as *B. mori* DHCR24 from now on. It was not possible to quantify rates of reaction or specific activity in this system, since the yeast extract is a crude enzyme preparation and it is impossible to determine how much of the protein content is *B. mori* DHCR24. The activities in the assays have, therefore, been expressed as a percentage of desmosterol converted into cholesterol compared to the empty vector (Fig. 4b).

### DNA encoding DHCR24 (desmosterol reductase)

The DHCR24 mRNA consists of an ORF of 1518 bp, that is predicted to encode a polypeptide of 506 amino acids, with a calculated molecular mass of 58.9 kDa. According to the current version of the *Bombyx mori* genome sequence database (http://silkworm.genomics.org.cn/), the gene spans ∼ 11 kb on scaffold 2836 and comprises 10 exons separated by 9 introns. The encoded protein has a predicted, conserved FAD-binding site (Fig. 2) and contains a potential N-terminal secretory signal sequence consistent with its microsomal localization (see later) but no predicted transmembrane helices.

### Cofactor requirement of *Bombyx* DHCR24

To determine the enzyme requirement for the cofactors NADPH and FAD, assays were carried out with various combinations of these (Fig. 4b). An assay supplemented neither with NADPH nor FAD produced a 25% conversion of desmosterol into cholesterol, whereas addition of NADPH alone (no FAD) produced a conversion almost as high (about 85%) as an assay with both cofactors added. Addition of FAD with the NADPH increases the conversion of desmosterol into cholesterol to 100% which indicates that this cofactor also stimulates the enzyme activity. However, the quantitative effect of FAD cannot be ascertained, since in its presence, the reaction has gone to completion in less than the 4 h incubation time and FAD has presumably already been loaded on to the enzyme by *S. cerevisiae*. Similarly, the fact that the assay with no cofactors still achieves significant conversion of the substrate into cholesterol is likely due to NADPH from the yeast extract itself, that is used up before complete conversion is achieved due to lack of the NADPH regenerating system.

### Tissue and sub-cellular association of DHCR24

**Figure 5:**
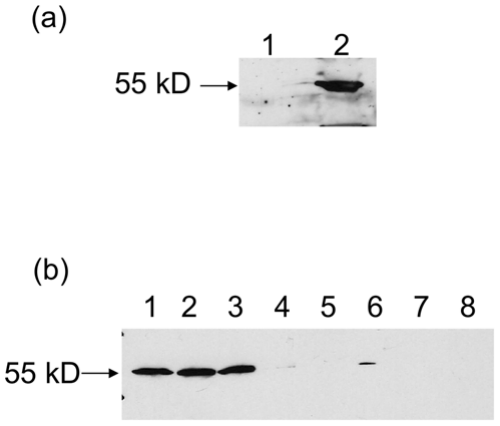
Western blot analysis of subcellular location and tissue distribution of *B. mori* DHCR24. (a) Samples of soluble supernatant (1) and microsomal (2) fractions, normalised for protein concentration were blotted with anti-DHCR24 antibodies. (b) Microsomes produced from various tissue homogenates normalised for protein concentration were probed with anti-DHCR24 antibodies: (1) foregut, (2) midgut, (3) hindgut, (4) testes, (5) ovary, (6) Malpighian tubules, (7) fat body, (8) head.

Once the DHCR24 activity had been confirmed, antibodies were raised to a peptide sequence toward the C-terminus of the protein and used to analyse the subcellular localisation and tissue distribution of the protein in *B. mori*. The protein was found to be present exclusively in the microsomal fraction of a midgut sample (Fig. 5a) suggesting specific membrane localisation. Samples of microsomes from various tissues (Fig. 5b) were also analysed by Western blot which revealed that the protein was present throughout the gut of the animal with a very small amount also detected in Malpighian tubules and traces in testes.

**Figure 6:**
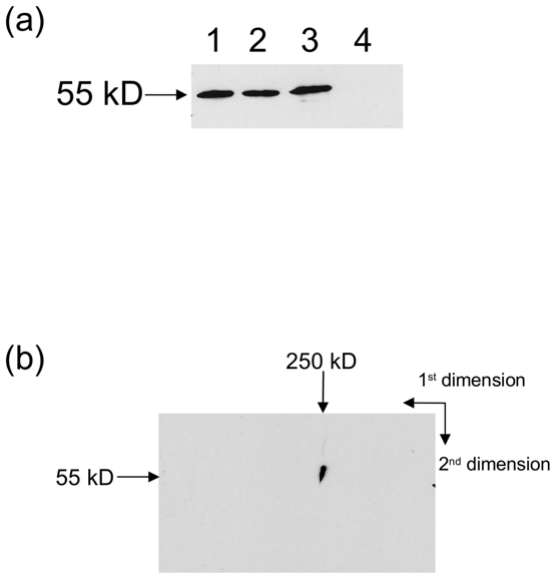
Membrane association and complex formation of *B. mori* DHCR24. (a) Equal aliquots of midgut microsomes were washed with 10 volumes of 5 mM HEPES/NaOH, pH 7.5 on ice for 30 min before reisolating the microsomes and analysing by western blot using anti-DHCR24 antibody. The washes were as follows: (1) HEPES/NaOH, pH 7.5 only, (2) HEPES/NaOH, pH 7.5+200 mM KCl, (3) HEPES/NaOH, pH 7.5+500 mM KCl, (4) 0.1 M Na_2_CO_3,_ pH 11.5. (b) Midgut microsomal protein was solubilised with 10% dodecyl maltoside and separated using native blue gel electrophoresis with native protein markers. This 1st dimension strip was then soaked in β-mercaptoethanol and SDS before running on a standard SDS PAGE gel and analysing by western blot using anti-DHCR24 antibody.

Since the protein was associated with the membrane fraction, high salt and alkaline Na_2_CO_3_ washes of the membrane fraction were performed to ascertain whether the protein can be separated from the membranes under these conditions (Fig. 6a). Alkaline sodium carbonate is known to remove peripherally associated membrane proteins [37]. The protein remained associated with the microsomal fraction after a 30 min treatment with 200 mM and 500 mM KCl, but was completely washed off by a similar treatment with alkaline Na_2_CO_3_. This indicates that the protein is peripherally associated with membrane rather than inserted into a membrane.

A two-dimensional native blue gel analysis of midgut microsomes was also carried out to determine whether the protein is associated with the membrane as a monomer or as part of a larger complex. Western blot analysis of the denaturing second dimension showed that the protein was part of a large complex with molecular weight approximately 250 kDa (Fig. 6b), which would suggest that the protein is either a multimer itself or associates with other proteins at the membrane. The predicted molecular weight of DHCR24 monomer protein is 58.9 kDa.

### Developmental expression of DHCR24 in midgut

**Figure 7:**
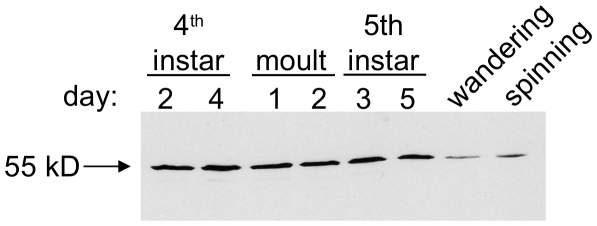
Expression of *B. mori* DHCR24 in midgut through the last two larval instars. Samples of midgut microsomes taken at regular intervals throughout the last two instars were normalised for protein concentration and analysed by western blot using anti-DHCR24 antibodies.

The expression of the protein in midgut microsomes throughout the last two larval instars was analysed by Western blot (Fig. 7). The protein is present at a fairly constant level throughout the last two instars, including the two-day moult period when the insects do not eat, but is drastically reduced during the wandering/spinning stages when the insects stop eating and expel gut contents in preparation for metamorphosis.

## Discussion

### Phylogenetic analysis

Using the more distantly related cytokinin dehydrogenase as an outgroup, phylogenetic analysis divides the sequences analysed into four distinct groups (Fig. 3). The first contains the *B. mori* DHCR24 identified here (BGIBMGA005735) and the human enzyme previously characterized. The second group contains the second *B. mori* sequence, BGIBMGA012624, the third consists of Arabidopsis DIMINUTO gene product and closely related sequences, while the fourth group consists of proteins from two *Meloidogyne* species. Sequence identities between the *Meloidogyne* sequences and groups 1–3 are low (28–30% with the group 3 plant sequences; 28–31% with group 2, and 30–36% with group 1). It is somewhat surprising that the *Caenorhabditis* sequences, CeF52H2.6 and CbCBG02085, that are derived from dealkylating species, show low affinity for group 1 in the tree (Fig. 3). However, the sequence identities of these genes justify including them in group 1∶34–36% identical vs. the plant group 3, 34–36% vs. the *Meloidogyne* group 4, 34–36% vs. group 2, and 43-48% vs. group 1. The sequences from two *Meloidogyne* plant–parasitic nematode species do not show affinity for those from the latter *Caenorhabditis* nematode sequences, a genus that dealkylates [6]. However, as alluded to in the Introduction, evidence for phytosterol dealkylation in *Meloidogyne* species is inconclusive.

It is likely that group 1 enzymes all catalyse the DHCR24 reaction although, as mentioned, for different ends. In the insect members of group 1, the DHCR24 reaction is the last in the pathway (dealkylation) that derives essential cholesterol from phytosterols present in the diet. Interestingly, these insect sequences are reliably grouped with those of the crustacean, *Daphnia pulex* and the plant pathogen protist, *Aphanomeyces euteiches*. Crustacea, like insects, are dependent on dietary sterols as a source of cholesterol [41] and sitosterol and desmosterol fully meet the dietary requirements of *Daphnia* [42]. In contrast, *Aphanomyces euteiches* can grow in minimal medium without exogenous sterols and DHCR24 presumably functions as part of a complete *de novo* cholesterol synthesis pathway [43]. The embedding of the biosynthetic *Aphanomyces euteiches* sequence in a clade otherwise composed of sterol transforming enzymes illustrates the facility with which the metabolic context of DHCR24 can change during evolution. Elsewhere in group 1, it has been reported that *Trichoplax adhaerens* contains a near-complete complement of sterol synthesis enzymes [44] and, thus, DHCR24 would be operating in biosynthetic mode. In contrast, the *C. elegans* protein, F52H2.6 and its *C. briggsae* counterpart, are presumably operating in conversion of dietary sterols into cholesterol since these worms are incapable of sterol synthesis [45].

For the plant enzymes, encoded by the *Arabidopsis* gene DIMINUTO and relatives, indirect evidence strongly suggests a role in the isomerisation and reduction of sterols, both the conversion of isofucosterol into sitosterol and the processing of 24-methylenechosterol into campsterol [46]. The reduction steps of both processes are highly similar to the reaction catalysed by DHCR24.

There are, as yet, no experimental data regarding the function of the proteins in group 2 but it is likely they catalyse the reduction and/or isomerisation of similar steroid substrates to those processed in groups 1 and 3. In sequence comparisons, group 1 DHCR24s share 34–42% identity with group 2 and 33–42% identity with group 3. The range for the comparison of groups 2 and 3 is 31–36%. One attractive possibility is that the enzymes in group 2 catalyse the first step of the pathway from sitosterol to cholesterol, namely sitosterol dehydrogenase (Fig. 1), catalysing the conversion of sitosterol into fucosterol. If this were the case, then a group 2 enzyme would be expected to invariably accompany a DHCR24 when the latter is employed for the processing of plant sterols, but not when operating in a biosynthetic capacity. Thus, a group 2 enzyme would be required for phytophagous insects but not those obtaining C_27_ sterols in the diet. As explained above, this would suggest that *C. elegans*, the plant pests, *T. castaneum, B. mori* and the crustacean, *D. pulex*, should have group 2 enzymes. This distribution is largely observed: group 2 sequences are found in the complete insect genomes from *T. castaneum, B. mori*, and in the *Daphnia* and *Caenorhabditis* genomes (Fig. 3), but surprisingly, not in the available genome of *A. aegptyi*, which dealkylates. It is not possible to be definitive about the absence of a group 2 enzyme in *Aphanomyces euteiches*, which would agree with the expected distribution, since that genome is not yet complete. However, there is no evidence whatsoever of a group 2 protein in the present genome resources [47].

### Alternative *Bombyx* DHCR24 candidate gene (BGIBMGA012624)

We also cloned and heterologously expressed in yeast, the second *Bombyx* DHCR24 candidate protein (BGIBMGA012624), but could not demonstrate DHCR24 activity (data not shown). Indeed, a recent microarray study in *Bombyx* [48] indicated that the RNA encoding this protein is, like the DHCR24 transcript, highly expressed in midgut, suggesting a role in a digestive/metabolic process. Thus, in line with the suggestion above that group 2 enzymes might catalyse desaturation of C-24 alkyl groups, we tested the enzymic activity of the expressed BGIBMGA012624. Unfortunately, we were unable to demonstrate any conversion of sitosterol into fucosterol, of campesterol into 24-methylene cholesterol, nor of cholesta-5,22,24-trien-3ß-ol into desmosterol with this protein using the same *S. cerevisiae* expression system as for BGIBMGA 005735. However, this sitosterol to fucosterol conversion reaction has been historically difficult to demonstrate *in vitro* even using insect tissue slices [2], so it may suggest specific requirements for enzymic activity that have as yet not been identified, possibly the formation of a complex with other proteins or perhaps the requirement for a sterol carrier protein system [49].

### DNA encoding DHCR24

Although the cDNA encoding DHCR24 in the dealkylation pathway from *Bombyx* is of a similar calculated molecular weight to the human biosynthetic enzyme (58.9 kDa for *Bombyx*, 60.1 kDa for human), the corresponding gene, spanning ∼11 kb in *Bombyx* is much more compact than the human one (∼46.4 kb). Furthermore, there is one more exon in *Bombyx* than in humans.

### Tissue and subcellular localisation

The Western blots of desmosterol reductase in *Bombyx mori* (Fig. 5b) show that the protein is almost entirely associated with the gut (foregut, midgut and hindgut), with a small amount in testes and Malpighian tubules. In previous studies on *B. mori*, fucosterol epoxide dealkylation activity was demonstrated in whole gut [50] and midgut [51] preparations, with significant activity outside the gut [50]. Furthermore, in *Spodoptera littoralis* (Lepidoptera; cotton leafworm), fucosterol dealkylation (total activity and specific activity) was far highest in midgut, with much less activity in hindgut and foregut, together with gonads, fat body, Malpighian tubules, and body wall [2], [52]. In the current work, no reductase protein was detected in fat body. Interestingly, since comparable amounts of desmosterol reductase protein were found in foregut, midgut and hindgut of *Bombyx*, if the midgut is the main site of dealkylation in this species, as in *Spodoptera*, the significance of the enzyme protein in the foregut and hindgut is unclear.

Western blotting of supernatant and microsomal fractions from *Bombyx mori* (Fig. 5a) showed localisation of the desmosterol reductase solely in the microsomal fraction. This corresponds to earlier work demonstrating fucosterol-24(28)-epoxide dealkylating activity primarily in the microsomal fraction in *Spodoptera littoralis* [2], [52]. Although subcellular localization of dealkylating activity has not been determined in *B. mori*, fucosterol-24(28)-epoxide dealkylation occurred as efficiently in the 25,000 g supernatant of gut as in the 1,500 g supernatant, consistent with a post-mitochondrial localization [50]. In line with the localisation of other sterol-metabolizing enzymes, the desmosterol reductase would be expected to be mainly in the smooth endoplasmic reticulum. As shown in Fig. 5a, the protein migrates in PAGE as a distinct band of approx. 55 kDa, in line with its theoretical molecular weight (58.9 kDa). This is similar to the molecular weight of the human protein (60.1 kDa).

Removal of the desmosterol reductase protein from the microsomes by alkaline sodium carbonate, indicates that the reductase protein is peripherally associated with the endoplasmic reticulum membrane. The human DHCR24 protein (also known as seladin-1) has also been localized to the endoplasmic reticulum membrane [53]. 2D-Gel analysis of midgut microsomal proteins by first dimension native blue gel fractionation and second dimension denaturing PAGE, followed by Western blot analysis (Fig. 6b), revealed that the reductase protein is part of a large complex (approx. 250 kDa). This suggests that the protein could either be a tetrameric protein (predicted molecular weight of a tetramer approx. 240 kDa), or is associated with other proteins, perhaps as part of a complex.

### Developmental expression

The reductase protein appears to be present at a constant level throughout the last two instars, including the two-day 4th/5th instar moult period when the larvae do not feed, but have a full gut (Fig.7). However, the enzyme protein level is much reduced during the wandering/spinning stages, when feeding stops and gut contents are purged. The developmental switch from feeding to wandering and gut purge is hormonally controlled, occurring when the titre of juvenile hormone is almost negligible, but with appearance of a small peak of ecdysteroid [54]. The sharp decline in desmosterol reductase protein levels at wandering could conceivably result from a hormonally-controlled developmental switch, such as the foregoing, that stops expression of this and other unnecessary processes for the pupal and adult stages at metamorphosis. These non-feeding stages have no need for a gut-associated digestive/metabolic process.

Interestingly, searches of TRANSFAC [34] revealed the presence of putative binding sites for the Broad Complex (BR-C) transcription factor [55] in the region upstream of the BGIBMGA005735 coding sequence. With a search configured to minimise prediction of both false positives and false negatives, five sites were predicted commencing, relative to the ATG codon from which protein translation starts, at −20, −551, −554, −659 and −942. The last of these is still predicted at more restrictive settings designed to minimise solely the number of false positive predictions and may therefore be considered particularly reliable. In contrast, analysis showed the absence of sites for binding of sterol regulatory element-binding protein (SREBP) [56], [57] upstream of BGIBMGA005735. Similar analysis of the region upstream of BGIBMGA012624 again reveals multiple putative Broad Complex-binding sites at −200, −569, −575 and −581 although none is predicted at the more stringent settings. Again, there is no putative site for SREBP. Although relatively short motifs such as transcription factor binding sites will be present by chance at a certain frequency in random DNA sequences, these results are at least consistent with Broad Complex controlling the expression of *B. mori* DHCR24 and, potentially, of the related protein BGIBMGA012624.

BR-C is an early ecdysteroid-induced transcription factor that is known to be required for induction of many late ecdysteroid-regulated genes. Although data are lacking on expression of BR-C in gut tissues of *Bombyx*, its expression increases in epidermis at the time of the small ecdysteroid peak [58], [59]. Similarly, BR-C expession in larval dorsal abdominal epidermis of *Manduca sexta* appears at the time of the small ecdysteroid peak that triggers wandering and continues for the rest of that stage [60]. Furthermore, BR-C is known to suppress transcription of larval genes [61]. Thus, it is quite conceivable that down-regulation of the DHCR24 gene, required during larval feeding stages, is mediated via the BR-C transcription factor. Owing to the apparent absence of an SREBP binding site in the upstream region of the DHCR24 gene, it is unlikely that transcription of the gene is sterol-regulated.

### Concluding remarks

It has been known for a long time that most invertebrates require a dietary source of sterols, many of which undergo processing to produce C_27_ sterols such as cholesterol. While the processing pathways are broadly mapped out, the identities of several of the enzymes responsible remain unknown. Here we fill one of those gaps by identifying and describing, for the first time, a DHCR24 acting in a phytosterol processing setting. The enzyme, from *Bombyx mori*, is homologous to mammalian sequences that catalyse the same reaction in an entirely different biochemical context, *de novo* cholesterol biosynthesis. Indeed, phylogenomic analysis shows the ease with which the enzyme switches roles during evolution. In agreement with previous data and its newly assigned biochemical role, *B. mori* DHCR24 is found in the microsomal cell fraction and is expressed predominantly in the gut. Furthermore, it is developmentally regulated and only expressed when larvae are feeding or have full guts. Putative Broad Complex transcription factor binding sites detectable upstream of the DHCR24 gene may play a role in its regulation.
